# Technical Note: An open source solution for improving TG‐263 compliance

**DOI:** 10.1002/acm2.12701

**Published:** 2019-09-19

**Authors:** Rex A. Cardan, Elizabeth L. Covington, Richard A. Popple

**Affiliations:** ^1^ Department of Radiation Oncology University of Alabama‐Birmingham Birmingham AL USA

**Keywords:** scripting, structure nomenclature, task group 263

## Abstract

**Purpose:**

Compliance with TG‐263 nomenclature standards can be challenging. We introduce an open source solution to this problem and evaluate its impact on compliance within our institution.

**Materials/methods:**

The TG‐236 nomenclature standards were implemented in our clinic in two phases. In phase 1, we deployed TG‐263 compliant templates for each disease site. In phase 2, we developed and deployed a script for evaluating compliance which presented errors to the user. After each phase the compliance was recorded.

**Results:**

Mean compliance errors prior to phase 1 was 31.8% ± 17.4%. Error rates dropped to 8.1% ± 12.2% across phase 1 and dropped further to 2.2% ± 6.9% during the automation system deployed in phase 2.

**Conclusion:**

Both structure templates and automation scripts are very useful for increasing compliance with structure naming standards. Our software solution is made available on GitHub for other institutions to implement.

## 1 INTRODUCTION

Standardized organ and target naming increases quality and safety within one's clinic and increases the ease of sharing data between clinical sites. As the need for sharing data increases, with clinical trials and outcomes research, the radiation oncology community has worked together to adopt a standard for organ and target naming, as well as dose volume histogram (DVH) metrics. This standard was recently detailed in American Association of Physicists in Medicine Task Group 263: Standardizing Nomenclatures in Radiation Oncology.^1^ To be inclusive of all aspects of radiation oncology, Task Group 263 membership comprised of radiation oncologists, clinical physicists, vendor representatives, dosimetrists, as well as members of NRC Oncology, RTOG, and IROC. This task group report includes two authors from our institution; consequently, we were early adopters of the standard. Throughout our implementation of the standard, we have tracked metrics on compliance and documented any roadblocks that our clinic has encountered.

This paper will focus on two methods that we have used to increase compliance with the TG‐263 nomenclature: (a) introduction of treatment planning system (TPS) templates, (b) release of an automated structure name checker within the TPS application program interface (API). Our automated checks are available as open source software on GitHub to facilitate the adoption of the standards at all radiation oncology clinics. We believe this is the first study to utilize automation to increase compliance of TG‐263 nomenclature standards. Through automation, we were able to significantly reduce the use of noncompliant name by making errors more visible to dosimetrists and physicists.

## 2 MATERIALS AND METHODS

This study was performed on clinical treatment plans developed in Eclipse (Varian Medical Systems, Palo Alto, CA). Compliance with TG‐263 nomenclature standards was studied over a 32‐month period. Phase 1 was approximately 18 months followed by 8 months in Phase 2. Compliance during the 6 months prior to Phase 1 is also presented.

### 2.1 Phase 1

Upon adoption of the TG‐263 standard, we released a departmental policy that all structure names were to be compliant with the standard. While recognizing that policies and procedures can be the least effective means of ensuring quality and safety,^2^ we concurrently released structure templates with‐in the TPS to facilitate compliance. Structure templates were created for each treatment site (e.g., head and neck, prostate). Additionally, a template with every structure name was created so physicians and dosimetrists could add structures as needed.

### 2.2 Phase 2

Approximately 18 months later, we released an automated structure name checker via an API script within the TPS. TG‐263 allows for multiple variations of structure names for a given anatomical location. To accommodate possible variations, a strict structure name dictionary was not used. Instead, a list of regular expressions was created which allowed for all possible compliant variations of a structure. Dosimetrists were instructed to run the checks and correct noncompliant names before physician plan approval.

Compliance on the use of each of these techniques was monitored during the previous phases using the scripting API. When it was noticed that compliance was decreasing, the dosimetrists and physicians were encouraged to continue the use of the tools during the planning process. They were also discouraged from creating structures without the use of templates. The number of errors for each plan was recorded and plans were binned into months for reporting. Compliance rates were averaged for each month of the 32 month study and compliance uncertainties were calculated across all the months in each phase.

## 3 RESULTS

There were 30 646 structure names and 29 631 structures names evaluated for phases 1 and 2, respectively. For a baseline comparison, the 6 months prior to phase 1 implementation were retrospectively analyzed for compliance. During this period of no specific TG‐263 guidance, the mean error percentage, calculated as the number of noncompliant structures divided by the total number of structures, was 31.8% ± 17.4%. Starting with policies and structure templates in phase 1, the mean dropped to 8.1% ± 12.2% over the 18‐month period monitored. Finally, with the introduction of automation checks in phase 2, in conjunction with policies and structure templates, the rate dropped to 2.2% ± 6.9%.

**Figure 1:**
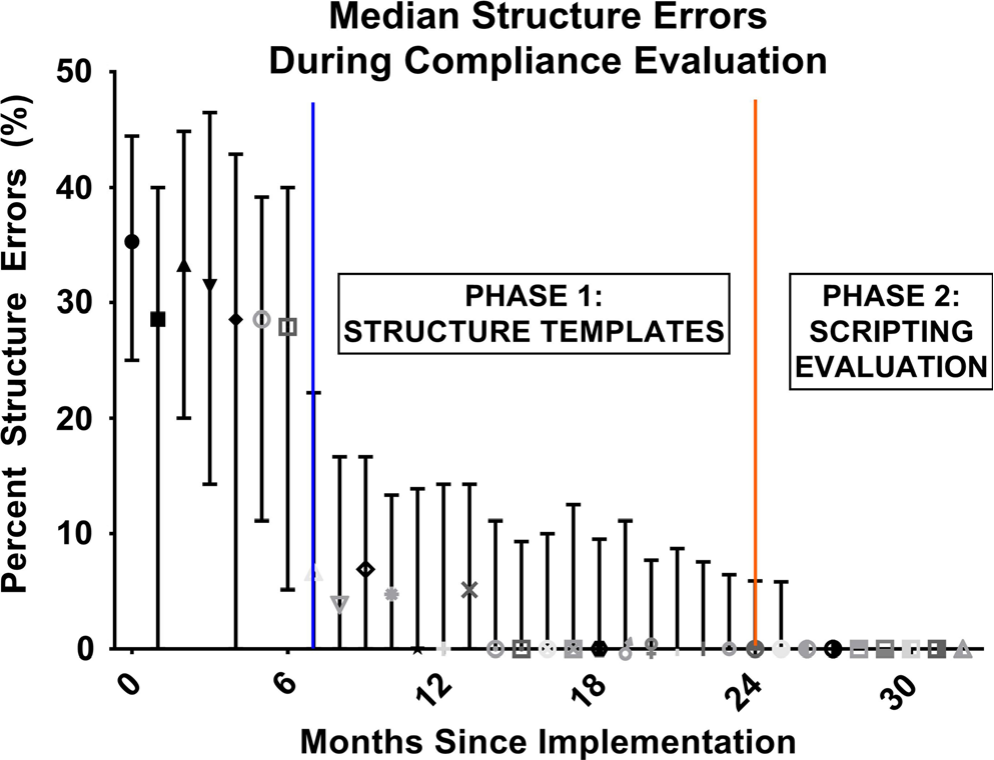
Median percent errors per month with interquartile range over the three time periods: no policies or TG:263 compliant templates (left), PHASE 1: policy and TG:263 compliant structure templates (middle), and PHASE 2: automated checking with scripting API (right).

The median values with quartile ranges observed by month are shown in Fig. 1. Upon the initialization of each phase, compliance tended to improve gradually. In the last 3 months observed, the median compliance rate dropped to 0.00% with a mean rate of 0.67% ± 2.76%.

### 3.1 Open source distribution

The authors merged the libraries with the open source C# Eclipse Scripting API Extension library (ESAPIX) on GitHub.com. The implementation was consolidated to a single method call for ease of use. The method *GetNameCompliance()* can be called from the class *TG263Dictionary* in just one line of code.

## 4 DISCUSSION

To our knowledge, there are no reports of automated tools to prospectively increase compliance with TG‐263 nomenclature standards. Schuler et. al reported on a tool, Stature, to retrospectively relabel structures in existing radiotherapy plans.^3^ This tool was also implemented in the TPS and required physician review of structure names to ensure correct mapping. Identification of structures is of great importance for outcomes related research, both retrospectively and moving forward. While TG‐263 has laid a solid framework for clinics, we have shown that it is not a trivial task to implement effectively. Beyond structure templates, the authors believe that automation systems should be put in place to verify compliance. To help aid clinics in getting started, we have developed an open source solution to this problem. Even though this particular implementation is designed to work with the Eclipse Scripting API, the open source distribution and multi‐platform C# language makes it ideal for inspection and modification to suite the user's needs.

We found that compliance with recommended naming standards can be very challenging, both technically and practically. To many staff members, the exact name of structures can seem trivial and unimportant to patient care. To mitigate this perception, the physicists had to continue to discuss the benefits of naming conventions both for single patients (DVH/plan quality analysis) and groups of patients (outcomes research).

## 5 CONCLUSION

We have demonstrated tools which have improved TG‐263 compliance in our center. Additionally, we have released the software toolkit open source on GitHub for other institutions to implement. It is hoped that these tools can be used for both future compliance and retrospective analysis of noncompliant structures. Future work includes identifying structures using information beyond the structure labels to increase automation capabilities and even higher compliance.

## CONFLICT OF INTEREST

The author serves as a contractor for Varian Medical Systems.
